# A comparison of phenotypic and WGS drug susceptibility testing in *Mycobacterium tuberculosis* isolates from the Republic of Korea

**DOI:** 10.1093/jacamr/dlad056

**Published:** 2023-05-13

**Authors:** Seung Heon Lee, Elena Ferran, Adam A Witney, Sungweon Ryu, Hyungseok Kang, Nathaniel Storey, Timothy D McHugh, Giovanni Satta

**Affiliations:** Division of Pulmonary, Sleep, and Critical Care Medicine, Department of Internal Medicine, Korea University Ansan Hospital, Korea University College of Medicine, Ansan, Korea; Barts Health NHS Trust, Pathology, London, UK; Institute for Infection and Immunity, St George’s University of London, London, UK; Clinical Research Centre, Masan National Tuberculosis Hospital, Changwon, South Korea; Clinical Research Centre, Masan National Tuberculosis Hospital, Changwon, South Korea; Great Ormond Street Hospital for Children NHS Foundation Trust, Microbiology, Virology and Infection Prevention and Control, London, UK; Centre for Clinical Microbiology, Department of Infection, University College London, London, UK; Centre for Clinical Microbiology, Department of Infection, University College London, London, UK

## Abstract

**Background:**

WGS has significant potential to help tackle the major public health problem of TB. The Republic of Korea has the third highest rates of TB of all Organisation for Economic Cooperation and Development countries but there has been very limited use of WGS in TB to date.

**Objectives:**

A retrospective comparison of *Mycobacterium tuberculosis* (MTB) clinical isolates from 2015 to 2017 from two centres in the Republic of Korea using WGS to compare phenotypic drug susceptibility testing (pDST) and WGS drug susceptibility predictions (WGS-DSP).

**Methods:**

Fifty-seven MTB isolates had DNA extracted and were sequenced using the Illumina HiSeq platform. The WGS analysis was performed using bwa mem, bcftools and IQ-Tree; resistance markers were identified using TB profiler. Phenotypic susceptibilities were carried out at the Supranational TB reference laboratory (Korean Institute of Tuberculosis).

**Results:**

For first-line antituberculous drugs concordance for rifampicin, isoniazid, pyrazinamide and ethambutol was 98.25%, 92.98%, 87.72% and 85.96%, respectively. The sensitivity of WGS-DSP compared with pDST for rifampicin, isoniazid, pyrazinamide and ethambutol was 97.30%, 92.11%, 78.95% and 95.65%, respectively. The specificity for these first-line antituberculous drugs was 100%, 94.74%, 92.11% and 79.41%, respectively. The sensitivity and specificity for second-line drugs ranged from 66.67% to 100%, and from 82.98% to 100%, respectively.

**Conclusions:**

This study confirms the potential role for WGS in drug susceptibility prediction, which would reduce turnaround times. However, further larger studies are needed to ensure current databases of drug resistance mutations are reflective of the TB present in the Republic of Korea.

## Introduction

TB, caused by *Mycobacterium tuberculosis* (MTB), resulted in 1.3 million deaths globally in 2020, and drug-resistant TB remains a public health emergency.^1^ The Republic of Korea has the third highest TB incidence and mortality rates of all Organisation for Economic Cooperation and Development (OECD) countries at 49 and 3.8 cases per 100 000, respectively, compared with 6.9 and 0.46 cases per 100 000 in the UK.^2^

Significant developments in the application of WGS in TB have enabled it to be used to aid outbreak investigations, epidemiological linkage of strains to monitor transmission, and identification of genes associated with both drug resistance and virulence factors.^3^ The identification by WGS of mutations associated with drug resistance is of growing interest because it allows a TB drug resistance profile to be ascertained in an average of 1 to 2 weeks from culture results as opposed to 6 to 8 weeks by current phenotypic drug susceptibility testing (pDST) methods as well as up to 7% lower cost.^4,5^

The potential future of WGS as the mainstay of DST is promising in settings where culture is not readily available, or a high risk of MDR-TB makes antitubercular drug selection time sensitive. More rapid results would allow patients with drug-resistant TB to be started on a personalized drug combination earlier, conceivably improving TB outcomes, reducing transmission and the risk of toxicity from unnecessary drugs.^6,7^ There is significant potential for WGS to be used for drug resistance testing in the Republic of Korea, which had a total of 629 and 47 cases of MDR and XDR TB, respectively, in 2020.^2^ In addition, the Republic of Korea has the laboratory capability and expertise to set up WGS testing at a national level as seen during the COVID-19 pandemic.^8^

Before WGS can be applied clinically it is necessary to demonstrate concordance between pDST and WGS drug susceptibility predictions (WGS-DSP) to an acceptable level as per WHO Delphi criteria.^9^ The CRyPTIC consortium was the largest comparison between phenotypic and WGS-DSP for first-line TB drugs; with 10 209 samples across 16 countries, it showed that the high sensitivity and specificity for these four drugs would allow for WGS-DSP to be applied clinically.^10^ However, there are currently no studies comparing pDST with WGS-DSP in the Republic of Korea, and local data for drug resistance mutations are needed because SNPs can vary between MTB lineages and may have implications for drug selection in some populations.^11^ For example, different lineages can have higher epidemiological cut-off values breakpoints for new drugs such as pretomanid.^12^ The majority of studies are so far in agreement that further research is needed to understand drug resistance mutations for second-line TB drugs.^13–15^

From an epidemiological perspective, WGS allows for the detailed monitoring of changes in drug susceptibility within a population, in addition to monitoring for outbreaks and transmission patterns, which can in turn inform an evidence-based screening programme. There are two population groups with the highest TB incidence in the Republic of Korea, the elderly and migrants from countries with high rates of TB. The patterns of migration and screening seem to have contributed to the higher incidence of TB including MDR in foreign-born migrants. In 2016 the Republic of Korea’s government required foreign-born migrants to have a TB-free certificate in order to apply for and extend a visa.^16^ This may have led to an increase in the number of patients identified with MDR-TB born outside of Korea, from 17 cases/year to 145 cases/year between 2011 and 2015.^17^ WGS would be able to identify if these are due to reactivation or newly acquired infection. By analysing a larger number of TB samples with WGS, the information may be used to further support public health measures for TB screening and management.

To date, WGS has been applied to MTB in the Republic of Korea in very small numbers; one study compared nine different strains of MTB between populations in the Republic of Korea, which found different lineages between TB from Chinese migrants and native Koreans.^18^ WGS has also been applied to investigate false positive results due to laboratory contamination.^19^ However, there have been no studies comparing genotypic predictions and phenotypic susceptibilities in MTB samples from the Republic of Korea to date. The current 2021 WHO catalogue of MTB complex mutations associated with drug resistance does not include any samples from the Republic of Korea.^20^ In view of the relatively high rates of TB the application of WGS for drug resistance prediction could be significantly impactful but it is important to study if the most current catalogue of resistance-conferring mutations applies to the TB circulating in the Republic of Korea.

This retrospective comparative study investigated the epidemiological links between MTB isolates from 2015 to 2017 using WGS, and compared pDST with WGS-DSP to antituberculous drugs from two TB centres, the Korean University An-San Hospital and the Masan National Tuberculosis Hospital, both in the Republic of Korea. The cohort of samples included fully drug susceptible, MDR, pre-XDR and XDR TB samples as per pre-2021 WHO definitions.^21^

## Methods

### M. tuberculosis isolates and setting

Clinical isolates were selected indiscriminately from two TB centres between 2015 and 2017, where both clinical details and microbiological samples were available. Half were native-born Koreans and the remainder were born outside the Republic of Korea. Korea University An-San Hospital is a large tertiary university hospital within Seoul National Capital Area, which treats around 300 patients with TB each year, of which 10% have MDR-TB. Masan National Tuberculosis Hospital is a tertiary referral hospital in the south of the peninsula and treats around 400 patients per year, of which 20% have MDR/XDR-TB. The former is a private hospital and the latter is a state-funded hospital serving a large population. The majority of patients are treated by a private-public mix model that includes TB specialist nurses and contact tracers. All TB diagnostics and treatment are government funded.^22^

The related clinical information was collected from medical chart reviews and inquiry to related facility or public health centres by S.H.L. in Korea University An-San Hospital and by H.S.K. in Masan National Tuberculosis Hospital and input into an anonymized Microsoft Excel spreadsheet.

### pDST

pDST was performed at the Korean Institute of Tuberculosis, Supranational TB Reference Laboratory, using the absolute concentration method in Löwenstein–Jensen medium observed with the following concentrations of TB drugs: isoniazid, 0.2 mg/mL; streptomycin, 10.0 mg/mL; ethambutol, 2.0 mg/mL; rifampicin, 40.0 mg/mL; para-aminosalicylic acid, 1.0 mg/mL; prothionamide, 40.0 mg/mL; cycloserine, 30.0 mg/mL; kanamycin, 30.0 mg/mL; capreomycin, 40.0 mg/mL; rifabutin, 20.0 mg/mL; ofloxacin, 4.0 mg/mL; levofloxacin, 2.0 mg/mL; moxifloxacin, 1.0 mg/mL; and linezolid, 2.0 mg/mL. Pyrazinamide susceptibility was determined using the pyrazinamidase test.^23^

### Extraction of genomic DNA and WGS

The clinical isolates were frozen at −80°C and DNA was later extracted in the Republic of Korea using the cetyltrimethylammonium bromide method.^3^ Extracted DNA underwent WGS at the National Mycobacterium Reference Service-South in the UK. WGS was carried out using the Illumina HiSeq platform (Illumina, San Diego, CA, USA). The DNA samples required a DNA 260/280 ratio of at least 1.8, with a concentration between 10 and 30 ng/mL.

### Bioinformatics and data analysis

WGS data were mapped to the H37Rv reference genome (RefSeq: NC_000962.3) using bwa mem (v0.7.17-r1188). Duplicates were removed and alignments sorted using samtools (v1.9). Sites were called using bcftools (v1.9) and filtered using the following criteria: mapping quality >30, site quality score >30, at least four reads covering each site with at least two reads mapping to each strand, and 75% of reads supporting the site (DP4).

Phylogenetic reconstruction was performed using IQ-Tree (v2.1.4) restricted to those models supported by raxml; branch support values were determined using 1000 bootstrap replicates. SNP distances were calculated using ancestral state reconstruction as implemented in pyjar.^24^ The WGS files were also mapped alongside the K strain, a previously published sequence of a common strain in the Republic of Korea.^25^ A larger tree was constructed including Korean isolate sequences available from the NCBI SRA database (accession PRJNA219826).

pDST was compared with the current known genotypic resistance mutations published on TB-Profiler, a TB-specific drug resistance prediction tool with a database of drug resistance-conferring mutations, on 2 March 2020. The sequences were screened for known mutations to 17 anti-TB drugs, including all first-line, second-line and two newer agents, bedaquiline and delamanid.

Further analysis was carried out using Microsoft Excel and SPSS Version 27 (IBM) to assess the concordance between phenotypic and genotypic resistance patterns, sensitivity (ratio between genotypic resistance and phenotypic resistance) and specificity (ratio between genotypically susceptible and phenotypically susceptible samples) of each drug and the *P* values using a paired sample *t*-test. The frequency of different resistance mutations was also analysed.

Finally, to investigate the possibility of previously unknown genetic resistance-conferring mutations the sequence reads were analysed using an in-house antimicrobial resistance (AMR) pipeline developed at Great Ormond Street Hospital and called ‘Rapid_treatR’. The AMR component of Rapid_treatR is based around the ARIBA tool (10.1099/mgen.0.000131) and utilizes a custom, manually curated AMR database based on the Comprehensive Antibiotic Resistance Database (see 10.1093/nar/gkz935) version 3.0.9 alongside in-house results processing scripts.

## Results

Fifty-seven clinical isolates cultured from the same number of patients were retrospectively collected. Thirty-seven cases were from Korea University An-San Hospital and 20 cases were from Masan National Tuberculosis Hospital. The median age was 48 years (16–92 years); 38 were male and 19 female. Forty-six had pulmonary TB, three extrapulmonary TB and eight had pulmonary TB combined with extrapulmonary TB. Table 1 shows further demographic data.

**Table 1.** Demographics of the 57 study patients

		Total, *n*	%
Gender	Male	38	66.7
	Female	19	33.3
Age (years)	Median (range)	48 (16–92)	
	11–30	6	10.5
	31–50	24	42.1
	51–70	21	36.8
	>71	6	10.5
HIV status	HIV positive	0	0.0
	HIV negative	57	100.0
Birthplace	Born in RoK	29	50.9
	Born outside RoK	28	49.1
Ethnicity	Korean	29	50.9
	Korean-Chinese	22	38.6
	Russian	3	5.3
	Uzbek	2	3.5
	Mongolian	1	1.8
Hospital	Korea University An-San Hospital	37	64.9
	Masan National Tuberculosis Hospital	20	35.1
Treatment	New treatment	34	59.6
	Retreatment	23	40.4
pDST classification	Fully susceptible TB	19	33.3
	MDR	19	33.3
	pre-XDR TB	11	19.3
	XDR TB	8	14.0
Site of disease	Pulmonary TB	46	80.7
	Extrapulmonary TB	11	19.3
	Pulmonary TB + extrapulmonary TB	9	15.79
Radiology—CT chest	Cavitating (suggestive of reactivation)	37	67.27
	Non-cavitating with atypical presentation (suggestive of primary infection)	18	32.73

RoK, Republic of Korea.

### Phylogenetic trees and lineages

**Figure 1.**
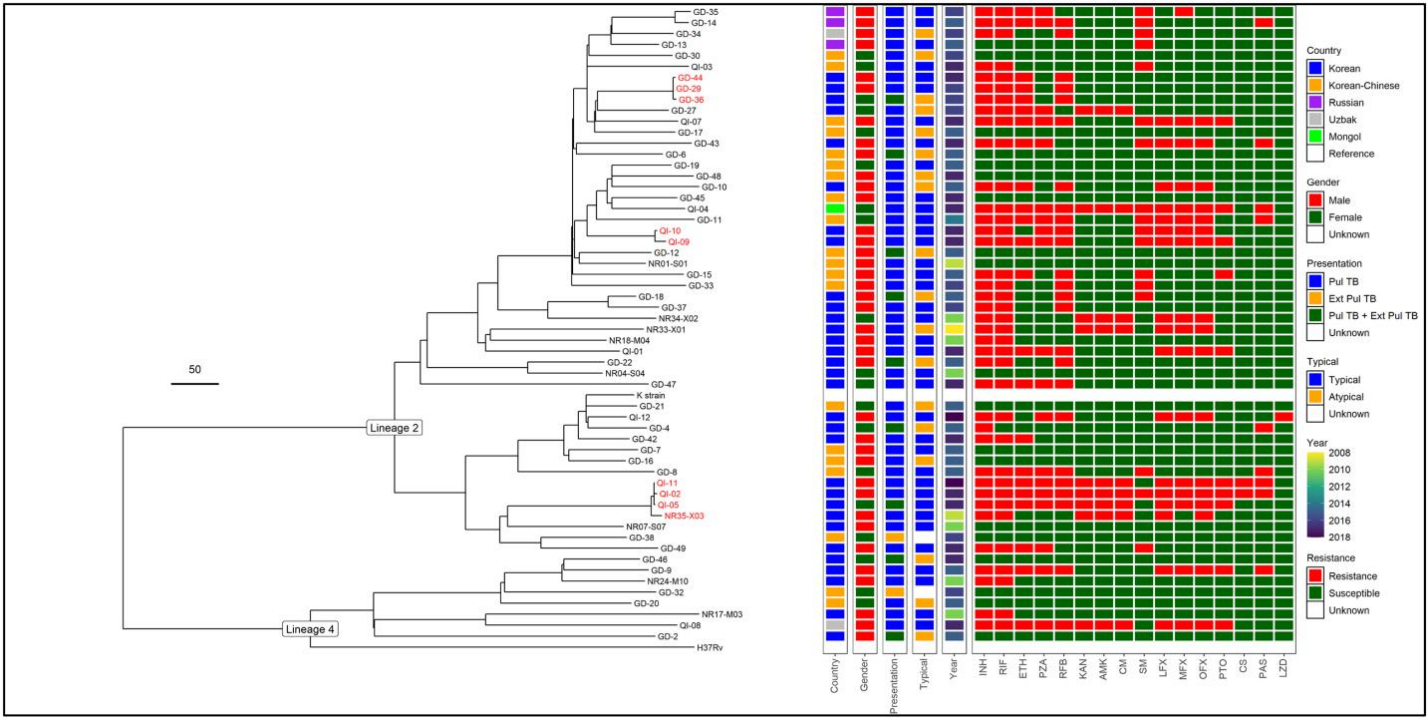
Tree A. Phylogenetic tree with the 57 sequence samples alongside reference sequence H37Rv and K-strain. Clusters are highlighted in red (defined as <12 SNPs difference). Each isolate is presented alongside pDST, country of birth, gender, presentation, typical versus atypical radiological findings, and year of diagnosis.

All isolates were successfully sequenced, and a phylogenetic tree constructed (Figure 1: Tree A). Forty-nine cases (86%) were lineage 2 (East Asian, including Beijing) and eight cases (14%) were lineage 4 (Euro-American).

There were three clusters of possible transmission amongst the patients, with an SNP difference of less than 12 as a cut-off.^26^

- *Cluster 1; GD-44/GD-29/GD-36*—The three patients lived in the same building. GD-44 and GD-29 were brothers who lived together, and GD-36 lived in an apartment in the same building. They all had had MDR-TB based on both phenotypic- and WGS-predicted DST.
- *Cluster 2; QI-09/QI-10*—These patients both had pre-XDR pulmonary TB and were treated at the same hospital on different days. Their SNP difference was only six SNPs therefore indicating possible recent transmission. No further information was available to understand an epidemiological link or direction of possible transmission.
- *Cluster 3; QI-11/QI-02/QI-05*—These isolates from three Korean patients from the same city with XDR-TB were new to treatment with typical cavitating pulmonary disease on imaging. No further information was available.

**Figure 2.**
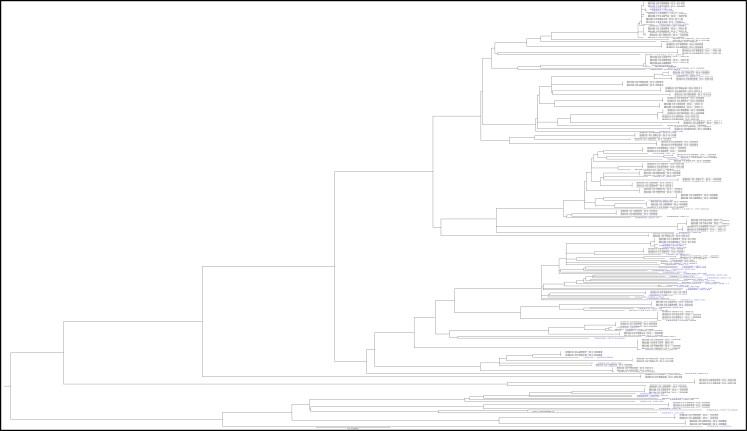
Phylogenetic tree with TB WGS from this study (in blue) alongside published TB WGS sequences from the Republic of Korea (in black) as per NCBI SRA database (accession PRJNA219826). Despite the limited number the selected strains are well distributed amongst the other national strains for which WGS is available.

In order to assess if the samples sequenced were representative of TB in the Republic of Korea, they were plotted alongside previously published TB genomes from the Republic of Korea on a larger phylogenetic tree, shown in Figure 2.^27^ There is an even distribution of the samples in this study amongst those previously published from the Republic of Korea.

### pDST and WGS-DSP concordance, sensitivity and specificity

By pDST, 19/57 samples were fully susceptible TB, 30/57 had MDR/pre-XDR TB, and 8/57 had XDR TB. From the pDST 14/38 (36.8%) patients with MDR/pre-XDR and XDR TB were born outside of South Korea.

**Figure 3.**
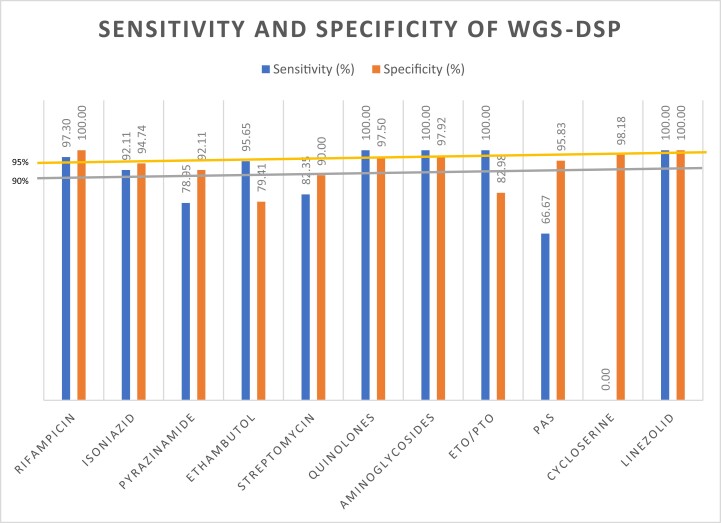
Sensitivity and specificity of WGS-DSP compared with pDST by antituberculous drug. ETO/PTO, ethionamide/prothionamide; PAS, para-aminosalicylic acid.

By comparing the pDST with the current known genotypic resistance mutations available in the TB-Profiler database on 2 March 2020 we were able to assess concordance between the two methods, as shown in Table 2. For first-line antituberculous drugs concordances for rifampicin, isoniazid, pyrazinamide and ethambutol were 98.25%, 92.98%, 87.72% and 85.96%, respectively. The sensitivity of WGS-DSP compared with pDST for rifampicin, isoniazid, pyrazinamide and ethambutol was 97.30%, 92.11%, 78.95% and 95.65%, respectively. The specificity for the first-line antituberculous drugs was 100%, 94.74%, 92.11% and 79.41%, respectively. The concordance for second-line drugs varied from 85.96% to 100%. The sensitivity ranged from 66.67% to 100% and specificity ranged from 82.98% to 100%. However, the overall numbers of samples that were resistant to cycloserine and linezolid were small. The sensitivities and specificities are summarized in Figure 3. There was statistical difference (*P* < 0.05) between pDST and WGS-DSP for ethambutol and ethionamide.

**Table 2.** Comparison of number of isolates that were resistant and susceptible to first- and second-line anti-TB drugs using pDST and WGS-DSP

	pDST		WGS-DSP		Concordance rate		Discordance rate		Sensitivity (%)	Specificity (%)	*P* value (*P* < 0.05)
Drug	R	S	R	S	Number	%	S versus R (%)	R versus S (%)			
Rifampicin	37	20	36	21	56	98.25	0.00	1.75	97.30	100.00	0.322
Isoniazid	38	19	36	21	53	92.98	1.75	5.26	92.11	94.74	0.322
Pyrazinamide	19	38	18	39	50	87.72	5.26	7.02	78.95	92.11	0.709
Ethambutol	23	34	29	28	49	85.96	12.28	1.75	95.65	79.41	0.033
Streptomycin	17	40	18	39	50	87.72	7.02	5.26	82.35	90.00	0.709
Quinolones	17	40	18	39	56	98.25	1.75	0.00	100.00	97.50	0.322
Aminoglycosides	9	48	10	47	56	98.25	1.75	0.00	100.00	97.92	0.322
ETO/PTO	10	47	18	39	49	85.96	14.04	0.00	100.00	82.98	0.004
PAS	9	48	8	49	52	91.23	3.51	5.26	66.67	95.83	0.659
Cycloserine	2	55	1	56	54	94.74	1.75	3.51	0.00	98.18	n/a^a^
Linezolid	1	56	1	56	57	100.00	0.00	0.00	100.00	100.00	n/a

ETO/PTO, ethionamide/prothionamide; PAS, para-aminosalicylic acid; R, resistant; S, susceptible.

a The *P* value was not calculated using *t*-tests for antibiotics with less than five samples.

### Analysis of mutations

**Figure 4.**
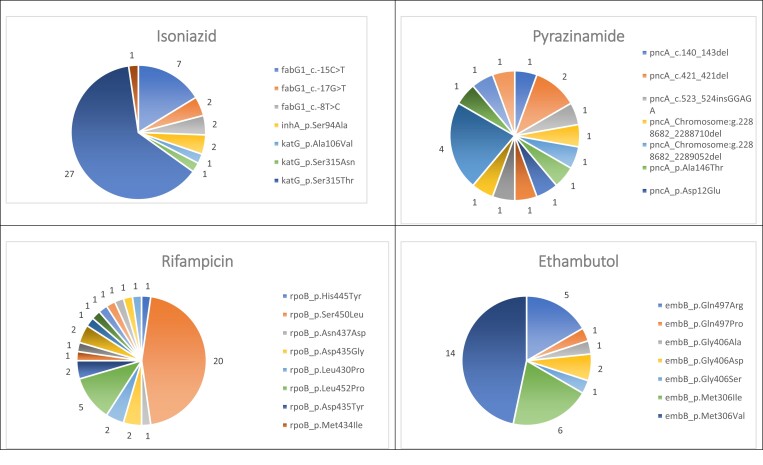
Frequency of first-line TB-drug resistance mutations.

The variation in the frequency of resistance is summarized in Figure 4. The most common mutation for rifampicin resistance was *rpoB*_Ser450Leu (20/44), for isoniazid resistance it was *katG*_Ser315Thr (27/43) and for ethambutol it was *embB*_Met306Val (14/30). Further information on frequency of mutations can be accessed in Table S1 (available as Supplementary data at *JAC-AMR* Online).

There were two samples (NR17-M03 and GD-27) that showed a discrepancy between the phenotypic and genotypic isoniazid resistance. NR17-M03 was phenotypically resistant to both rifampicin and isoniazid but genotypically only resistant to rifampicin, and GD-27 was phenotypically resistant to all the first-line drugs (rifampicin, isoniazid, pyrazinamide and ethambutol) yet genotypically predicted to be resistant to all but isoniazid. This raised the possibility of a false negative susceptible genotypic prediction for isoniazid because rifampicin resistance often develops after isoniazid resistance. These two isolates were further analysed by matching them to the reference strain (H37Rv) showing further non-synonymous mutations in the *katG* gene. NR17-M03 had mutation *katG_*Arg249Leu and GD-27 had mutations *katG*_Arg463Leu and *katG*_Val284Asp. There were no non-synonymous mutations detected in the *inhA* gene.

There were no genotypic resistance markers known to be associated with bedaquiline or delamanid identified in this cohort of isolates. Phenotypic resistance to bedaquiline and delamanid was not tested in these samples.

## Discussion

This study retrospectively analysed 57 MTB clinical isolates from the Republic of Korea following WGS for epidemiological links between them, to ensure they were representative of MTB isolates in the Republic of Korea, and compared the WGS-DSP with the pDST results.

The 57 samples were from two different hospitals, which receive the referred patients from the western and southern regions of the Korean peninsula, respectively. Of the three clusters identified by WGS, one had clear epidemiological links because two patients were brothers and the third lived in the same building. Unfortunately, there was no further clear information to understand the link between the other two clusters due to the retrospective nature of the study. Our findings would support use of WGS in South Korea as a useful public health tool to monitor outbreaks and reduce transmission as previously demonstrated in other countries.^3,7^

Overall, the samples integrated well with those previously sequenced from the Republic of Korea, showing that our cohort was representative at the national level. This is useful epidemiologically and for the next part of the study looking at WGS to predict drug resistance in the Republic of Korea but it does not reflect transmission. A further larger TB molecular epidemiology study with thorough epidemiological information including clinical data could help design an evidence-based TB transmission monitoring strategy. The majority of the 57 samples were lineage 2 (Beijing lineage), like the K strain, and consistent with the literature that lineage 2 is the dominant MTB lineage in the Republic of Korea. The Beijing lineage is associated with higher rates of drug resistance, increased risk of exogenous reinfection and transmissibility. This may be a microbiological factor that contributes to the high ranking of TB incidence in the Republic of Korea compared with other OECD countries.^25,28^

The samples tested in this study included a range of sensitivities and classifications from fully susceptible to MDR and XDR TB. The definitions used for assigning MDR, pre-XDR and XDR TB were pre-2021 WHO definitions because the samples were taken prior to 2021. Genotypic drug susceptibility was predicted by the WGS sequence applied through an online software tool, TB-profiler. This database was chosen due to good results in similar studies in Thailand and China^29,30^ and better concordance of drug susceptibility predictions compared with other online databases.^31^

The concordance between the pDST and the WGS-DSP methods met the WHO Delphi criteria for the first-line TB drugs rifampicin and isoniazid and some of the second-line agents such as aminoglycosides and quinolones. The sensitivity and specificity between WGS-DSP and pDST for rifampicin and isoniazid were similar to other studies.^32^ The high sensitivity for the fluoroquinolones and aminoglycosides was higher than other studies and the WHO catalogue, which had a pooled sensitivity of 84.4% for levofloxacin, 87.7% for moxifloxacin and 77.3% for amikacin. The 100% concordance for linezolid is due to the limited number included (*n* = 1).

The sensitivity for pyrazinamide and specificity for ethambutol were lower at 78.95% and 79.41%, respectively. Having a lower specificity for ethambutol is a common theme in similar studies form Thailand, Brazil, Mozambique, Sweden and China.^29,30,33,34^ Reasons for this include the difficulty in reproducing resistance testing phenotypically, uncertain breakpoints and incomplete understanding of resistance-conferring mutations.^35^ The low sensitivity for pyrazinamide is also commonly reported, and some similarly designed studies have excluded pyrazinamide from the comparison between WGS-DSP and pDST from the outset.^29,36^ This may be in part due to the difficulty in the pDST methods for pyrazinamide because it is only active at low pH 5.5, which can itself inhibit mycobacterial growth. Therefore, there can be a falsely high resistance rate. The genetic resistance mechanism is not fully understood for pyrazinamide and may not lend itself to the automated way the resistance databases work.^37,38^

Compared with a study from Thailand our most frequent mutations coincided for isoniazid, ethambutol, streptomycin, fluoroquinolones, ethionamide and two of the aminoglycosides, amikacin and capreomycin.^29^ The most common rifampicin resistance mutation found differed between the studies: the most common rifampicin mutation, *rpoB*_Ser450Leu in this study, did not feature in the samples from Thailand or China despite using the same database, i.e. TB-Profiler.^29,30^ This exemplifies the importance of evaluating the resistance-conferring mutations at a local level because these can vary.

With regard to second-line agents, aminoglycoside resistance was conferred by a single mutation (rrs_r.1401a > g) with no variation between the different aminoglycoside antibiotics; this differs from findings from Thailand, China and Sweden, which often had different resistance mutations and would not necessarily cancel out a whole antibiotic class.^4,29,30^ For the quinolones there were a variety of different mutations associated with resistance yet they each conferred resistance to the entire class rather than selected quinolones. Considering the introduction in South Korea of new drugs such as linezolid, bedaquiline and delamanid, early establishment of a genetic molecular test based on WGS would be helpful for early detection of any emerging resistance. Because the numbers in this study are low no further conclusions can be made for second-line agents.

Reasons for discrepancies between WGS-DSP predictions and pDST include an incomplete library of genetic resistance mutations, especially with second-line antituberculosis drugs,^29^ and difficulty with phenotypic testing for certain antibiotics such as pyrazinamide and ethambutol, as described above, including uncertainty of clinical breakpoints.^39^ There is also incomplete understanding of how certain mutations influence resistance.^15^ There are certain resistance mechanisms that would not be detected genotypically, such as efflux pump activations, which are most likely regulated through post-translational protein modification.^5^

The two samples investigated for new potential mutations conferring isoniazid resistance have found non-synonymous mutations in *katG* and were not present in TB-profiler. The WHO catalogue of resistance mutations Supplementary material showed that *katG_*Arg463Leu is not associated with resistance and is also associated with *Mycobacterium bovis* as a phylogenetic SNP. But this mutation is not exclusive to *M. bovis* and has been identified in proven cases of *M. tuberculosis*, as in this case.^40^*katG_*Val284Asp and *katG*_Arg249Leu are not present in the WHO catalogue, therefore it is possible that isoniazid resistance is being conferred by these two mutations and future work may show them to be significant in MTB isolates from the Republic of Korea.^20^ The WHO catalogue looked at 38 125 isolates from 41 countries but none of these were from the Republic of Korea.^20^ The Republic of Korea has a relatively higher incidence of mono-resistance to isoniazid (10% of all strains). Further analysis for isoniazid resistance mutations in more samples may help to find additional molecular targets responsible for drug resistance.^41^

Finally, limitations of this study include the relatively limited number of samples tested from only two hospitals, the retrospective nature of the analysis (rather than prospective and assessing the clinical impact) and the limited conclusions for second-line drugs.

Larger studies are required to ensure the resistance mutation databases encompass the resistance-conferring mutations seen in the Republic of Korea and are valid. Considering the updated WHO definition of pre-XDR and XDR since 2021 and the wider use of newer drugs, validation of WGS results for drugs such as linezolid, bedaquiline, delamanid and pretomanid should be undertaken using worldwide data.^42^ In addition, comprehensive and high-confidence genetic markers that translate into phenotypic resistance are necessary to distinguish resistant variants with clinical importance from those where the mutation is not conferring significant changes in terms of antibiotic susceptibility.

### Conclusion

This is the first study to compare WGS predictions for drug susceptibility in the Republic of Korea and affirms that WGS in TB is a valid public health tool for monitoring transmission and outbreaks. There is good preliminary evidence that WGS would be suitable for DST in the Republic of Korea for first-line drugs, but more work is needed for second-line drugs with a larger volume of samples.

This one technological tool applied at a national level would reduce turnaround times for drug susceptibility predictions, enhance outbreak monitoring and potentially reduce cost overall.

## Supplementary Material

dlad056_Supplementary_Data

## Data Availability

The data for this study have been deposited in the European Nucleotide Archive (ENA) at EMBL-EBI under accession number PRJEB51057 (https://www.ebi.ac.uk/ena/browser/view/PRJEB51057).

## Supplementary data

Table S1 is available as Supplementary data at *JAC-AMR* Online.
