# Supplementary material for: A comparison of phenotypic and WGS drug susceptibility testing in Mycobacterium tuberculosis isolates from the Republic of Korea
Source: JAC Antimicrob Resist. 2023 May 13;5(3):dlad056. doi: 10.1093/jacamr/dlad056 (PMC10182733; doi:10.1093/jacamr/dlad056)
Supplement: dlad056_Supplementary_Data [file dlad056_supplementary_data.docx]

**Supplementary**

Table S1: Frequency of 1^st^ and 2^nd^ line TB-drug resistance mutations

| Drug | Gene Muation | Frequency | Percentage |
| --- | --- | --- | --- |
| Rifampicin | rpoB_p.His445Tyr | 1 | 2.3 |
|  | rpoB_p.Ser450Leu | 20 | 45.5 |
|  | rpoB_p.Asn437Asp | 1 | 2.3 |
|  | rpoB_p.Asp435Gly | 2 | 4.5 |
|  | rpoB_p.Leu430Pro | 2 | 4.5 |
|  | rpoB_p.Leu452Pro | 5 | 11.4 |
|  | rpoB_p.Asp435Tyr | 2 | 4.5 |
|  | rpoB_p.Met434Ile | 1 | 2.3 |
|  | rpoB_p.Gln432Pro | 1 | 2.3 |
|  | rpoB_p.Asp435Tyr | 2 | 4.5 |
|  | rpoB_p.Ser441Leu | 1 | 2.3 |
|  | rpoC_p.Leu527Val | 1 | 2.3 |
|  | rpoB_p.Ser450Val | 1 | 2.3 |
|  | rpoB_c.1295_1303del | 1 | 2.3 |
|  | rpoB_p.Asp435Val | 1 | 2.3 |
|  | rpoB_p.His445Leu | 1 | 2.3 |
|  | rpoB_p.His445Arg | 1 | 2.3 |
| Isoniazid | fabG1_c.-15C>T | 7 | 16.3 |
|  | fabG1_c.-17G>T | 2 | 4.7 |
|  | fabG1_c.-8T>C | 2 | 4.7 |
|  | inhA_p.Ser94Ala | 2 | 4.7 |
|  | katG_p.Ala106Val | 1 | 2.3 |
|  | katG_p.Ser315Asn | 1 | 2.3 |
|  | katG_p.Ser315Thr | 27 | 62.8 |
|  | katG_p.Thr394Ala | 1 | 2.3 |
| Pyrazinamide | pncA_c.140_143del | 1 | 5.6 |
|  | pncA_c.421_421del | 2 | 11.1 |
|  | pncA_c.523_524insGGAGA | 1 | 5.6 |
|  | pncA_Chromosome:g.2288682_2288710del | 1 | 5.6 |
|  | pncA_Chromosome:g.2288682_2289052del | 1 | 5.6 |
|  | pncA_p.Ala146Thr | 1 | 5.6 |
|  | pncA_p.Asp12Glu | 1 | 5.6 |
|  | pncA_p.Leu151Ser | 1 | 5.6 |
|  | pncA_p.Leu85Arg | 1 | 5.6 |
|  | pncA_p.Ser67Pro | 1 | 5.6 |
|  | pncA_p.Thr135Pro | 4 | 22.2 |
|  | pncA_p.Thr76Pro | 1 | 5.6 |
|  | pncA_p.Trp68Cys | 1 | 5.6 |
|  | pncA_p.Val155Gly | 1 | 5.6 |
| Ethambutol | embB_p.Gln497Arg | 5 | 16.7 |
|  | embB_p.Gln497Pro | 1 | 3.3 |
|  | embB_p.Gly406Ala | 1 | 3.3 |
|  | embB_p.Gly406Asp | 2 | 6.7 |
|  | embB_p.Gly406Ser | 1 | 3.3 |
|  | embB_p.Met306Ile | 6 | 20.0 |
|  | embB_p.Met306Val | 14 | 46.7 |

2^nd^ line TB drugs

| Drug | Gene muation | Frequency | Percentage |
| --- | --- | --- | --- |
| Streptomycin | gid_c.115_115del | 1 | 5.6 |
|  | rpsL_p.Lys43Arg | 14 | 77.8 |
|  | rpsL_p.Lys88Arg | 1 | 5.6 |
|  | rrs_r.514a>c | 2 | 11.1 |
| Quinolones | gyrA_p.Ala90Val | 4 | 18.2 |
|  | gyrA_p.Asp94Ala | 4 | 18.2 |
|  | gyrA_p.Asp94Asn | 1 | 4.5 |
|  | gyrA_p.Asp94Gly | 11 | 50.0 |
|  | gyrA_p.Ser91Pro | 1 | 4.5 |
|  | gyrB_p.Glu501Asp | 1 | 4.5 |
| Aminoglycosides | rrs_r.1401a>g | 10 | 100.0 |
| prothionamide | ethA_c.1299_1300insG | 1 | 4.8 |
|  | ethA_c.364_365insA | 1 | 4.8 |
|  | ethA_c.820_830del | 3 | 14.3 |
|  | ethA_c.821_831del | 1 | 4.8 |
|  | ethA_Chromosome:g.4326004_4326574del | 1 | 4.8 |
|  | ethA_Chromosome:g.4326086_4327547del | 1 | 4.8 |
|  | fabG1_c.-15C>T | 7 | 33.3 |
|  | fabG1_c.-17G>T | 2 | 9.5 |
|  | fabG1_c.-8T>C | 2 | 9.5 |
|  | inhA_p.Ser94Ala | 2 | 9.5 |
| PAS | folC_p.Ser150Gly | 1 | 12.5 |
|  | thyA_p.Thr22Ala | 1 | 12.5 |
|  | thyX_c.-16C>T | 6 | 75.0 |
| CS | ald_c.460_460del | 1 | 100.0 |
| Linezolid | rrl_r.2814g>t | 1 | 100.0 |
